# 

**Published:** 1895-06

**Authors:** 


### CONTENTS.

ORIGINAL ARTICLES—

Wounds of the Eye and Their Treatment. By T. E. Mitchell, M. D., Columbus, Ga.......................................... 291

Spectacle-Glasses: When to be Worn and How They Should be Selected. By A. Bethune Patterson, M. D., Atlanta, Ga....... 298

Clinical Notes on a Recent Series of Surgical Cases. By Thomas H. Manley, M. D., New York............................... 304

### CONTENTS.—Continued.

SOCIETY REPORTS—

Maryland Clinical Society .................................... 321
Gynecological and Obstetrical Society of Baltimore ........... 325

SELECTIONS AND ABSTRACTS—

Rational Therapeutics of Cholera Infantum .................... 331

EDITORIAL—

Announcement ................................................. 333
Special Notes ................................................ 336
Prescriptions ................................................ 340

